# Supplementary material for: In Situ Compatibilized Blends of PLA/PCL/CAB Melt-Blown Films with High Elongation: Investigation of Miscibility, Morphology, Crystallinity and Modelling
Source: Polymers (Basel). 2023 Jan 6;15(2):303. doi: 10.3390/polym15020303 (PMC9864367; doi:10.3390/polym15020303)

Table S1. Compositions and tensile property of ternary-blended, melt-blown films of PLA/PCL/CAB samples

Samples	PLA/PCL/CAB Compositions	% Elongation at break [MPa]		Tensile strength at break [MPa]		Modulus [MPa]	
		MD	TD	MD	TD	MD	TD
1	100PLA	19.44	10.31	45.02	44.57	2780	2800
2	95PLA/5PCL	16.93	14.82	50.29	45.23	3104	3014
3	90PLA/10PCL	26.19	9.88	41.59	42.74	2848	2880
4	80PLA/20PCL	12.71	1.07	37.28	11.11	2677	1968
5	95PLA/5CAB	35.88	10.52	52.94	49.78	3145	3070
6	90PLA/10CAB	13.84	30.86	53.89	48.36	3157	2942
7	80PLA/20CAB	5.44	3.15	43.17	58.48	2557	2590
8	90/5/5	20.03	3.41	39.94	41.53	1344	1471
9	85/10/5	19.04	4.33	35.55	32.58	2282	2366
10	85/5/10	358.73	318.81	47.42	44.31	2452	2310
11	80/15/5	26.68	2.98	29.83	27.55	2263	1935
12	80/10/10	4.57	1.82	35.45	33.72	2107	2094
13	80/5/15	279.21	288.60	38.52	44.18	2182	2300
14	75/20/5	25.91	1.65	26.64	19.26	1895	1678
15	75/10/15	353.79	245.45	39.33	36.18	2108	2162
16	75/15/10	364.08	4.61	36.53	37.04	2163	2213
17	75/5/20	3.23	3.02	48.72	50.45	2112	2272
18	70/20/10	2.72	1.87	36.23	39.21	2334	2342
19	70/15/15	391.85	7.04	44.20	27.43	2227	2102
20	70/10/20	12.30	1.97	32.40	30.09	1940	1812
21	65/25/10	5.87	0.58	8.41	2.42	564.8	471
22	65/20/15	274.85	0.61	36.10	9.43	2194	1715
23	65/15/20	349.49	10.65	34.89	24.08	1812	1592
24	65/10/25	196.66	186.13	42.43	49.84	2707	2877
25	60/25/15	6.69	0.78	22.11	8.68	1490	1256
26	60/20/20	5.28	1.79	29.22	2.16	1917	422.5
27	60/15/25	395.57	9.64	44.08	29.98	2126	2010
28	55/20/25	421.71	9.33	40.63	18.61	1965	1202
29	50/25/25	3.10	3.30	29.21	23.99	1764	1634
30	50/20/30	28.13	2.52	20.13	15.36	1346	908.8

Figure S2a. 1H NMR of ternary-blended, melt-blown films of HE and LE films comparing with the solvent casted-films, showing zoom peaks at chemical shift of 3.4-3.8 ppm.

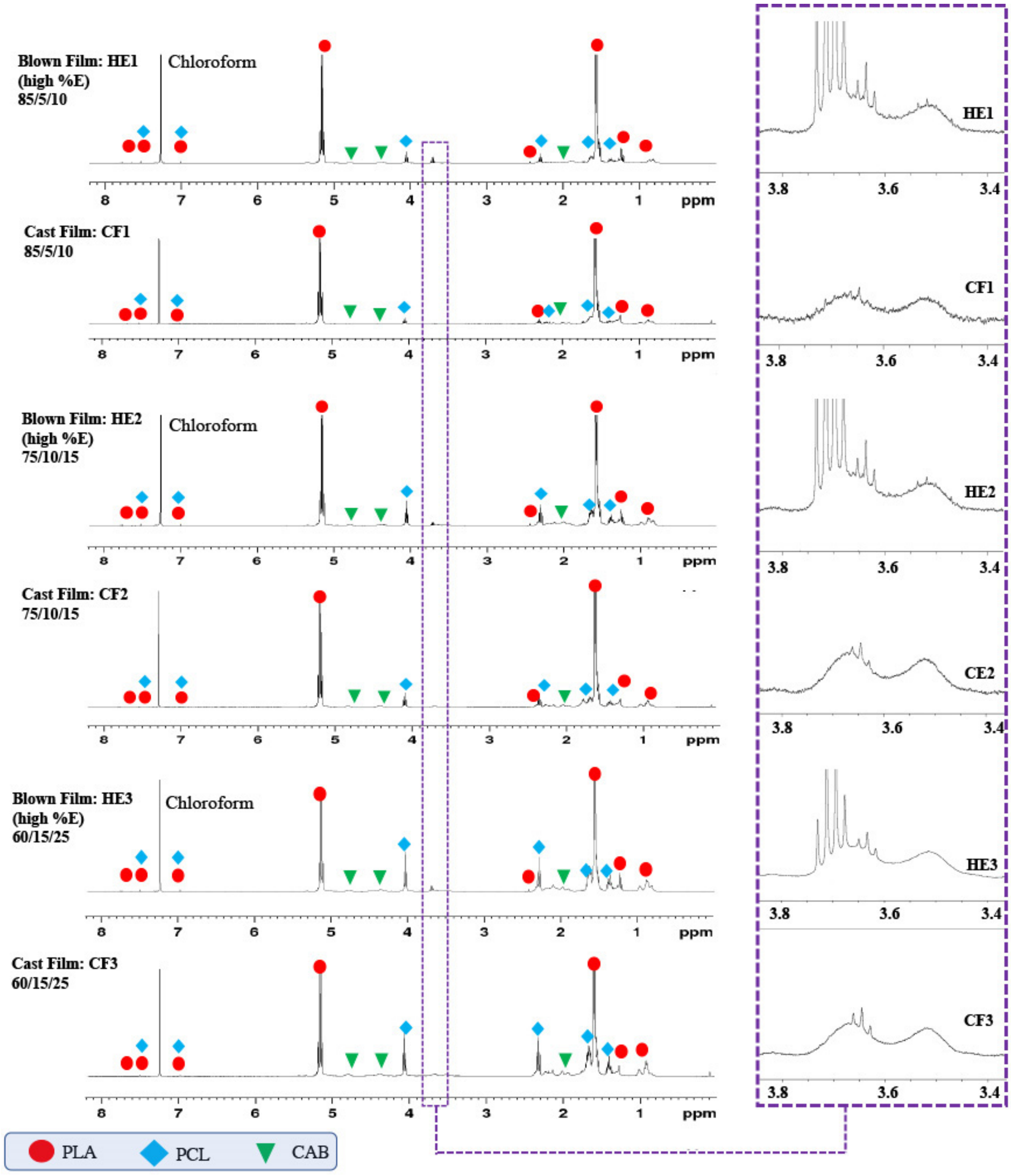

Figure S2b. 13C NMR of ternary-blended, melt-blown films of HE and LE films comparing with the solvent casted-films, showing zoom peaks at chemical shift of 58.4-58.8 ppm ppm.

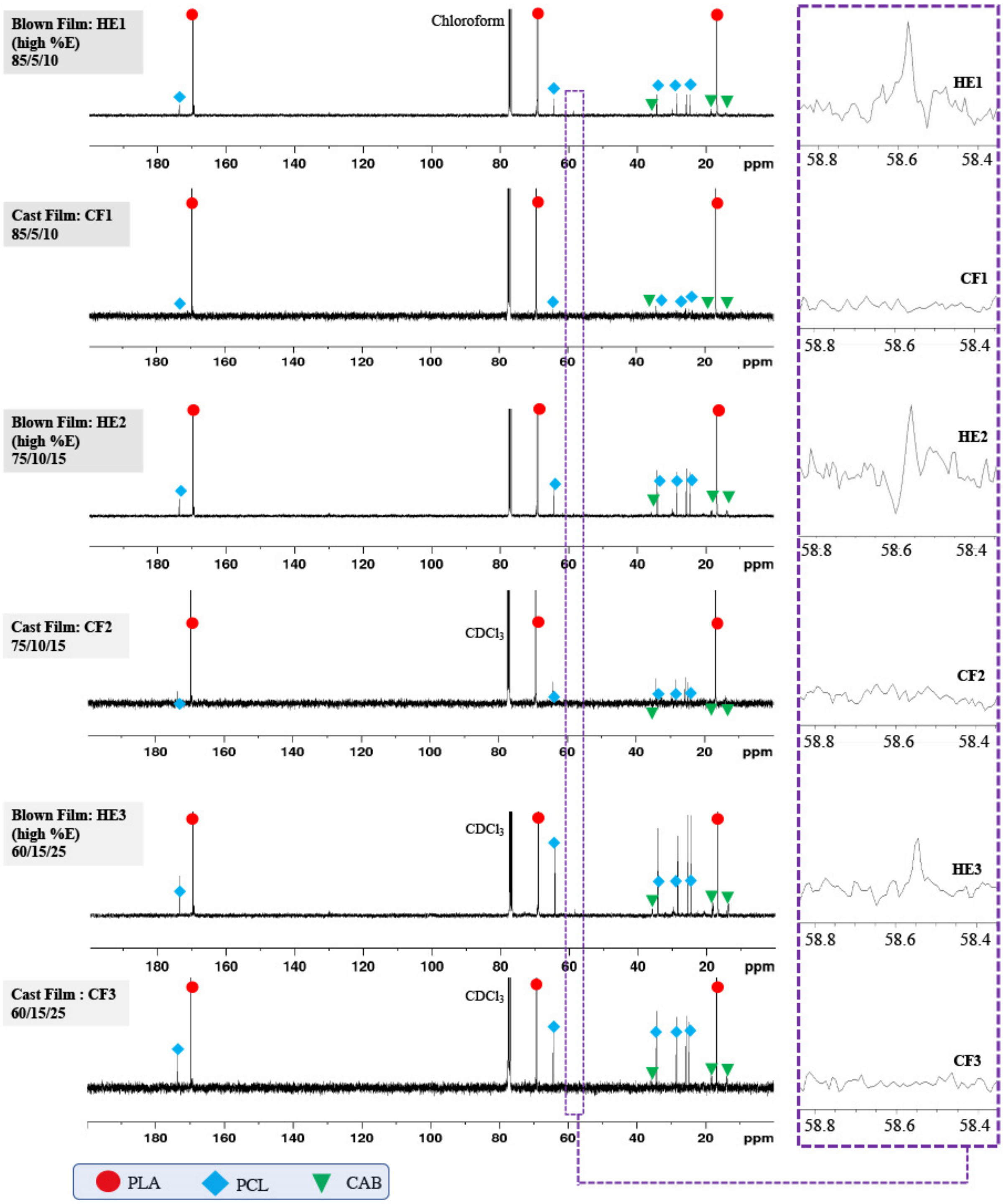

Supplement: Supplementary file 1 [file polymers-15-00303-s001.zip › polymers-2121521-SM.pdf]
